# Adhesion Molecules in Lung Inflammation from Repeated Glyphosate Exposures

**DOI:** 10.3390/ijerph20085484

**Published:** 2023-04-12

**Authors:** Upkardeep Pandher, Shelley Kirychuk, David Schneberger, Brooke Thompson, Gurpreet Aulakh, R. S. Sethi, Baljit Singh

**Affiliations:** 1 Health Sciences Graduate Program, University of Saskatchewan, 107 Wiggins Road, P.O. Box 23, Saskatoon, SK S7N 5E5, Canada; 2 Canadian Centre for Health and Safety in Agriculture, University of Saskatchewan, 104 Clinic Place, P.O. Box 23, Saskatoon, SK S7N 2Z4, Canada; 3 Department of Medicine, College of Medicine, Canadian Centre for Health and Safety in Agriculture, University of Saskatchewan, 104 Clinic Place, P.O. Box 23, Saskatoon, SK S7N 2Z4, Canada; 4 Department of Small Animal Clinical Sciences, Western College of Veterinary Medicine, University of Saskatchewan, 52 Campus Drive, P.O. Box 23, Saskatoon, SK S7N 5B4, Canada; 5 College of Animal Biotechnology, Guru Angad Dev Veterinary and Animal Sciences University, Ludhiana 141004, India; 6 Department of Veterinary Biomedical Sciences, Western College of Veterinary Medicine, University of Saskatchewan, 52 Campus Drive, P.O. Box 23, Saskatoon, SK S7N 5B4, Canada

**Keywords:** glyphosate, lung inflammation, repetitive exposure, adhesion markers

## Abstract

Glyphosate is an active ingredient in herbicides. Exposure to glyphosate-based herbicides has been associated with respiratory dysfunctions in agricultural workers. The ability of inhaled glyphosate to induce lung inflammation is not well understood. Further, the role of adhesion molecules in glyphosate-induced lung inflammation has not been studied. We evaluated lung inflammatory responses from single and repeated glyphosate exposures. Male C57BL/6 mice were intranasally exposed to glyphosate (1 μg/40 μL) for 1 day or once daily for 5 days or 10 days. Lung tissue and bronchoalveolar lavage (BAL) fluid were collected and analyzed. Repeated exposure to glyphosate for 5 days and 10 days resulted in an increase in neutrophils in BAL fluid and higher eosinophil peroxidase levels in lungs, with leukocyte infiltration further confirmed through lung histology. Repetitive exposure to glyphosate increased IL-33 and Th2 cytokines IL-5 and IL-13. A single glyphosate treatment revealed expression for ICAM-1, VCAM-1, and vWF adhesion molecules in the perivascular region of lung sections; with repeated treatment (5 and 10 days), adhesion molecule expression was found in the perivascular, peribronchiolar, and alveolar regions of the lungs. Repetitive exposure to glyphosate induced cellular inflammation in which adhesion molecules may be important to the lung inflammatory process.

## 1. Introduction

Glyphosate [N-(phosphonomethyl) glycine] is the most common active ingredient in herbicides. Glyphosate-based herbicides are extensively used in agriculture worldwide [1]. Glyphosate has been detected in air collected from farms during glyphosate application [2] and in urine samples of agricultural workers and family members [3,4,5]. Glyphosate exposure has been associated with respiratory effects in exposed workers [6,7,8,9,10,11].

There is little information on the ability of inhaled glyphosate to induce airway inflammation.. Recently, Kumar and colleagues challenged female C57BL/6 mice intranasally with different doses of glyphosate (100 ng, 1 μg or 100 μg) for 7 days and did not observe dose-dependent effects of glyphosate on markers of airway inflammation and lung pathology [2]. Glyphosate exposure for 7 days induced an increase in neutrophils in bronchoalveolar lavage (BAL) fluid, eosinophils in lungs, and IL-5 (BAL fluid), IL-13, IL-10, IL-33, and TSLP cytokines in blood samples of challenged mice [2].

In inflammation, a leukocyte-endothelial interaction is often initiated by chemotactic factors or cytokines, which stimulate adhesion through the activation of either leukocytes or endothelial cells. Further, a cellular immune response requires white blood cell accumulation, movement of the cell through the vessel wall, and tissue effect. This inflammatory cascade is regulated by adhesion molecules including intercellular adhesion molecule (ICAM-1) and vascular cell adhesion molecule (VCAM-1) from the immunoglobulin supergene family [12,13,14]. The von Willebrand factor (vWF) is an emerging mediator of vascular inflammation that is stored in platelets and endothelial cells [14]. Although the release of cytokines has been shown after glyphosate exposure, there is no data on the expression of adhesion molecules in the lungs of glyphosate-exposed animals.

Adhesion molecules have been shown to be involved in airway inflammatory effects in asthma and allergic rhinitis [15,16], two common conditions of workers exposed to glyphosate. LPS is a common inflammatory agent in agricultural exposures and strong expression of ICAM-1 and VCAM-1 has been found in lungs after LPS exposures [17,18,19,20], as well as after exposure to a combination of LPS and glyphosate [21,22]. Further, antibody blocking of ICAM-1 and VCAM-1 expression has been shown to reduce cellular immune response in the lungs [23,24,25], suggesting a role for adhesion molecules in lung inflammation. The role of adhesion molecules in inflammatory processes from repeated exposures to glyphosate may be important in unravelling lung inflammatory responses. The present study characterized lung inflammation and the expression of cytokines and adhesion molecules in the lungs of mice after single and repetitive inhalation treatments to glyphosate.

## 2. Materials and Methods

### 2.1. Mice Exposure

The experimental protocols were approved by the Animal Ethics Research Board of the University of Saskatchewan (Protocol # 20160106). Male C57BL/6 mice (Charles River Laboratories, Montreal, QC, Canada), 6–8 weeks old, were maintained at the Laboratory Animal Services Unit of the University of Saskatchewan. Mice were fed ad *libitum* and were acclimatized for one week after arrival.

Mice were divided into glyphosate and control treatment groups (n = 5 per group). The stock solution of glyphosate (0.8 M; analytical grade PESTANAL standard, Sigma, St. Louis, MO, USA) was prepared in Hank’s Balanced Salt Solution (HBSS; without calcium, pH 7.4, Life Technologies, Grand Island, NY, USA). It was vortexed for 10 min and syringe filtered (0.22 µm; Fisher Scientific, Waltham, MA, USA). Mice received 40 µL of either glyphosate (1 µg/40 µL) or HBSS (control group) intranasally for 1 day or daily for 5 days or 10 days. Mice were lightly anesthetized using isoflurane before treatments. A 1 µg dose of glyphosate was selected for inhalation treatment based on glyphosate levels found in the agricultural environment and because this was a level that had been utilized in other studies [2,21]. Four hours after the last treatment, mice were euthanized by CO_2_ inhalation and BAL fluid and lungs were collected.

### 2.2. Bronchoalveolar Lavage Fluid Collection and Processing

BAL fluid was collected by washing the airways three times with 0.5 mL of ice-cold HBSS. The collected BAL fluid was centrifuged at 1000 g for 10 min at 4 °C and supernatants were stored at −80 °C for analysis. Cells from BAL fluid were resuspended in HBSS and kept on ice until used for leukocyte counts. Total and differential leukocyte counts were performed using a hemocytometer and cytospin stained with a Protocol Hema 3 kit (ThermoFisher Scientific, Waltham, MA, USA). Cytokines were measured using a Custom Mouse Procartaplex Multiplex Immunoassay (ThermoFisher Scientific, Waltham, MA, USA), according to the manufacturer’s instructions for magnetic bead-based ELISA. Plates were read using a Bioplex 200 system (Bio-Rad, Mississauga, ON, Canada) and Bioplex Manager Software (Bio-Rad, Mississauga, ON, Canada).

### 2.3. Lung Tissue Collection and Processing

Following BAL fluid collection, the right lung was tied off at the primary bronchus and the left lung was fixed in-situ through intratracheal instillation of 200 µL of 4% paraformaldehyde (PFA). The right lung was removed, snap-frozen in liquid nitrogen, and stored at −80 °C and was used for eosinophil peroxidase (EPO) and RNA analysis. The fixative-instilled left lung was further submerged in 4% PFA for 16 h at 4 °C. Lung tissue was then washed through ascending grades of alcohol before embedding in paraffin. Lung sections of 5 µm thickness were cut from paraffin-embedded tissues on an American Optical Rotary Microtome (Model 820, American Optical, Buffalo, NY, USA) and placed onto pre-charged slides (ThermoFisher Scientific, Waltham, MA, USA). Hematoxylin & eosin staining, and immunohistochemistry were performed on lung sections.

### 2.4. Eosinophil Peroxidase Quantification

Lung tissues were homogenized using 2 mm Zirconia beads (BioSpec, Bartlesville, OK, USA) in tubes containing RIPA lysis buffer supplemented with 1X Halt Protease and Phosphatase Inhibitor Cocktail (ThermoFisher Scientific, Waltham, MA, USA) in a Mini- Beadbeater-24 homogenizer (BioSpec, Bartlesville, OK, USA) for two 1-min rounds. The tubes were cooled on ice in between rounds of homogenization. The total protein concentration of lung homogenates was determined using Pierce BCA Protein Assay Kit (ThermoFisher Scientific, Waltham, MA, USA) according to the manufacturer’s instructions. Eosinophil peroxidase (EPO) was quantified using a Mouse EPO DuoSet ELISA (LifeSpan Biosciences, Seattle, WA, USA). Plates were read using a BioTek Synergy HT plate reader (BioTek, Winooski, VT, USA) at 450 nm.

### 2.5. RNA Isolation and Real-Time PCR

Lung homogenates were prepared using 2 mm Zirconia beads (BioSpec, Bartlesville, OK, USA) in tubes containing RLT lysis buffer (Qiagen, Chatsworth, CA, USA) in a Mini-Beadbeater-24 homogenizer (BioSpec, Bartlesville, OK, USA). RNA was extracted using the RNeasy Plus Mini Kit (Qiagen, Chatsworth, CA, USA) according to the manufacturer’s instructions. Purified mRNA was quantified using a Take3 plate and a BioTek Synergy HT plate reader (BioTek, Winooski, VT, USA). cDNA was generated using iScript Reverse Transcription Supermix (Bio-Rad, Hercules, CA, USA) with 0.5 µg mRNA. PCR was conducted in a CFX96 Touch Real-Time PCR Detection System (Bio-Rad, Hercules, CA, USA) using the following protocol: 25 °C for 5 min, 46 °C for 20 min, and 95 °C for 1 min.

Real-time PCR was performed using probes for mouse ICAM-1 (Mm00516023_m1), TLR-4 (Mm00445273_m1), TLR-2 (Mm00442346_m1) (Life Technologies, Grand Island, NY, USA). Each reaction was carried out in duplicate using ribosomal RNA (Life Technologies, Grand Island, NY, USA) as an endogenous control. PCR was conducted in a CFX96 Touch Real-Time PCR Detection System (Bio-Rad, Hercules, CA, USA). PCR reactions were carried out as follows: 50 °C for 2 min, 95 °C for 10 min followed by 40 cycles at 95 °C for 15 s and 60 °C for 1 min. Relative quantification was estimated from each target gene’s cycle threshold, obtained from real-time PCR data, followed by analysis with the ΔΔCt method.

### 2.6. Histology and Scoring for Inflammation

Lung sections from all groups were stained with hematoxylin & eosin stain and mounted using Surgipath MM24 Mounting Media (Leica Biosystems, Richmond, IL, USA) before analysis. The detailed scoring criteria for cellular lung inflammation was described previously [22]. Briefly, the parameters scored were cellular infiltration (for polymorphonuclear cells and/or monomorphonuculear cells) in the perivascular, peribronchiolar, and alveolar regions, levels of alveolar thickness, and perivascular space. Each scored on a scale of 0 to 3. Scoring was performed under a 40× objective lens on five fields per section from each of the treated mice (N = 5 per treatment group). Four investigators independently, and blinded, scored the sections and an average lung histology score was then calculated for each parameter.

### 2.7. Immunohistochemistry and Analysis

Lung sections from mice were stained with antibodies against ICAM-1, VCAM-1, and vWF markers by following a previously described protocol [22]. Briefly, lung sections were immersed in a series of xylene baths for deparaffinization and in different alcohol grades for rehydration. Endogenous peroxidase activity was quenched with 0.5% hydrogen peroxide in methanol for 20 min. Antigen unmasking and blocking were done for 30 min with 2 mg/mL pepsin and 1% bovine serum albumin, respectively. The lung sections were incubated overnight at 4 °C with the following primary antibodies: ICAM-1 (dilution 1:100; rabbit monoclonal anti-mouse ICAM-1, ab179707, Abcam Inc., ON, Canada), VCAM-1 (dilution 1:100; rabbit monoclonal anti-mouse VCAM-1, ab134047, Abcam Inc., ON, Canada), and vWF (dilution 1:200; rabbit monoclonal anti-mouse vWF, ThermoFisher Scientific, Waltham, MA, USA). Following overnight incubation, the secondary goat anti-rabbit antibody (dilution 1:200; ThermoFisher Scientific, Waltham, MA, USA) was added onto tissue sections. Slides were incubated for 1 h at room temperature in a humidified chamber. The color was developed using a peroxidase kit (Vector laboratories, Burlington, ON, Canada) according to the manufacturer’s instructions and counterstained with methyl green (Vector laboratories, Burlington ON, Canada). In the end, slides were dehydrated through a series of ethanol concentrations and were fixed with xylene before mounting. Controls with the omission of the primary antibody or secondary antibody were run at the same time.

The expressions of ICAM-1, VCAM-1, and vWF were reviewed in five random fields of each lung section. Expression of ICAM-1 was scored in the vasculature and bronchial epithelium as described previously [22]. Briefly, each parameter was given a score based on staining intensity (0 = no or occasional staining, 1 = weak staining, 2 = moderate staining, 3 = intense staining).

### 2.8. Data Analysis and Statistics

Data was analyzed using GraphPad Prism 6 (GraphPad Software, San Diego, CA, USA). Error bars represent mean +/− SD. For values outside the assay limit of detection, a value was designated using either the lowest limit of detection (LLOD/2) or a value below the lowest attained value for the variable. Statistical significance was determined using one-way ANOVA with a follow-up Tukey test for multiple comparisons. If the assumption of equal variance was not met, the data was log-transformed, followed by either one-way ANOVA and multiple comparison tests or by non-parametric Kruskal-Wallace tests. A *p*-value < 0.05 was considered significant for differences between groups.

For graphing of data, “a” indicates a significant difference compared with the respective control group; “1” indicates a significant difference compared with the 1-day exposure group; “2” indicates a significant difference compared with the 5-days exposure group.

## 3. Results

### 3.1. Leukocyte Counts in BAL

**Figure 1:**
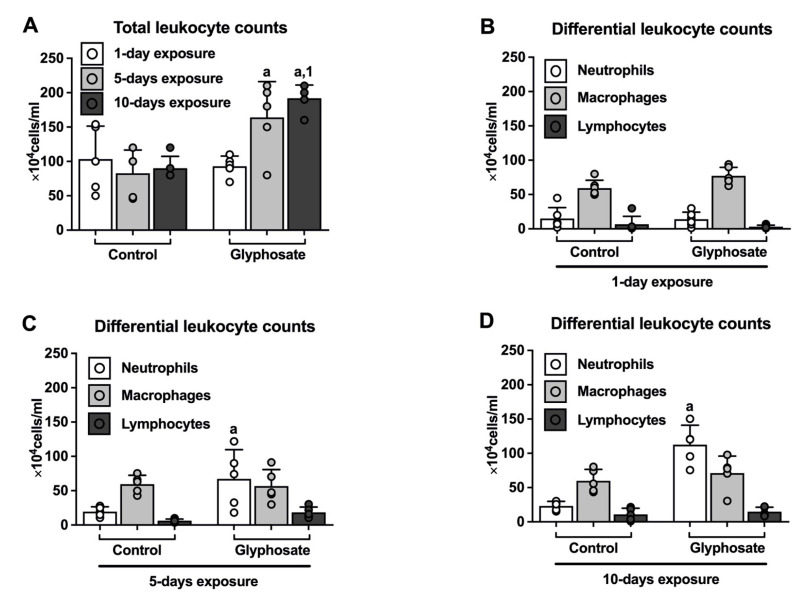
Leukocyte counts in bronchoalveolar lavage fluid. Total leukocyte counts (**A**) and differential leukocyte counts (**B**–**D**) were measured in the BAL fluid of mice after control or glyphosate treatment for 1 day, 5 days, and 10 days. Data presented as mean ± SD (N = 5 mice per group). Significance (*p* < 0.05) is denoted as such: “a” indicates a significant difference compared with the control group; “1” indicates a significant difference compared with 1-day treatment group.

Total leukocyte counts in BAL fluid were significantly higher after both 5 and 10 days of glyphosate treatment as compared to control exposures (Figure 1A) and total leukocyte counts after 10 days of glyphosate treatment were significantly higher as compared to the 1-day glyphosate treatment group.

Neutrophils were significantly higher after glyphosate treatments for both 5 days and 10 days as compared to same days of the control treatments; however, the total number of neutrophils after 1 day of glyphosate treatment were similar to the 1-day control treatment group (Figure 1B–D). The number of neutrophils increased in a stepwise fashion such that after 10 days of treatment with glyphosate, the number of neutrophils were almost double that of the 5-day glyphosate treatment group. Macrophage and lymphocyte counts in the glyphosate treatment groups were not significantly different than the control treatment groups (Figure 1B–D).

### 3.2. Cytokine Levels in BAL

**Figure 2:**
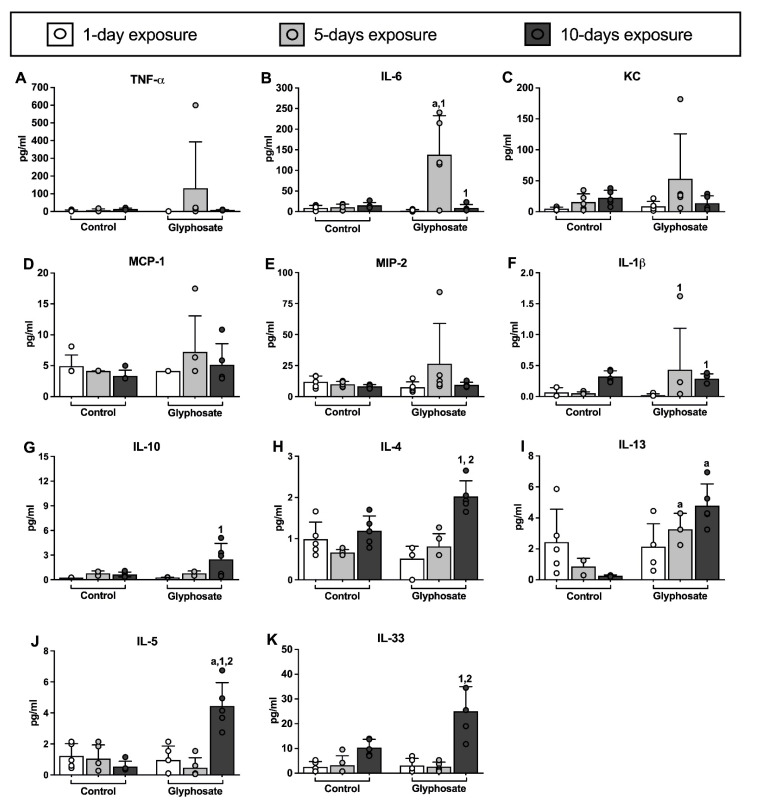
Cytokine levels in bronchoalveolar lavage fluid. Cytokines were measured in supernatant of BAL fluid collected from mice after treatment with saline (control) or glyphosate for 1 day, 5 days, and 10 days (**A**–**K**). Data presented as mean ± SD (N = 5 mice per group). “a” indicates a significant difference (*p* < 0.05) compared with the control group; “1” indicates a significant difference compared with the 1-day treatment group; “2” indicates a significant difference compared with the 5-days treatment group.

IL-6 (Figure 2B) and IL-1β (Figure 2F) both showed an increase after 5 days of glyphosate treatment and were back to levels similar to the control treatment after 10 days of glyphosate treatment. IL-10 (Figure 2G), IL-4 (Figure 2H), IL-5 (Figure 2J), and IL-33 (Figure 2K) were similar to the control treatment groups after 1 day and 5 days of glyphosate treatment, but all were significantly higher after 10 days of glyphosate treatment when compared to the 1-day glyphosate treatment group. The 10-day glyphosate treatment group showed significantly higher levels in IL-4, IL-5, and IL-33 compared to the 5-day glyphosate treatment group. IL-13 (Figure 2I) levels were similar to the control after 1 day of glyphosate treatment but were progressively higher with increasing days of glyphosate treatment and were significantly higher than the control treatment levels after both 5 days and 10 days of glyphosate treatment. There were no significant differences in TNF-α (Figure 2A), KC (Figure 2C), MCP-1 (Figure 2D), MIP-2 (Figure 2E) levels between 1 day, 5 days, and 10 days of glyphosate treatment and their respective control exposures.

### 3.3. Eosinophil Peroxidase in Lungs

**Figure 3:**
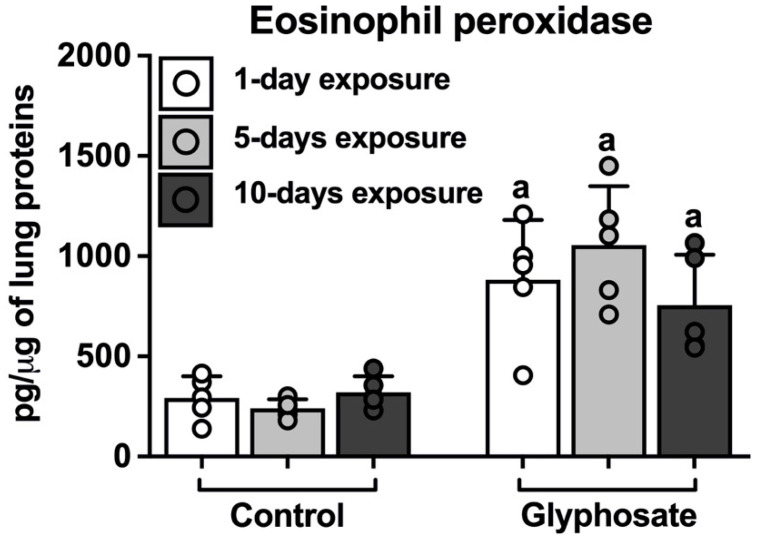
Eosinophil peroxidase levels in lungs. Eosinophil peroxidase (EPO) levels were measured in lungs of mice after exposure to saline (control) or glyphosate for 1 day, 5 days, and 10 days. Data presented as mean ± SD (N = 5 mice per group). “a” indicates a significant difference (*p* < 0.05) compared with the respective control group.

Glyphosate treatment significantly increased the EPO levels in the lungs after 1 day, 5 days, and 10 days of glyphosate treatments as compared to the respective control (Figure 3).

### 3.4. Lung Histology

**Figure 4:**
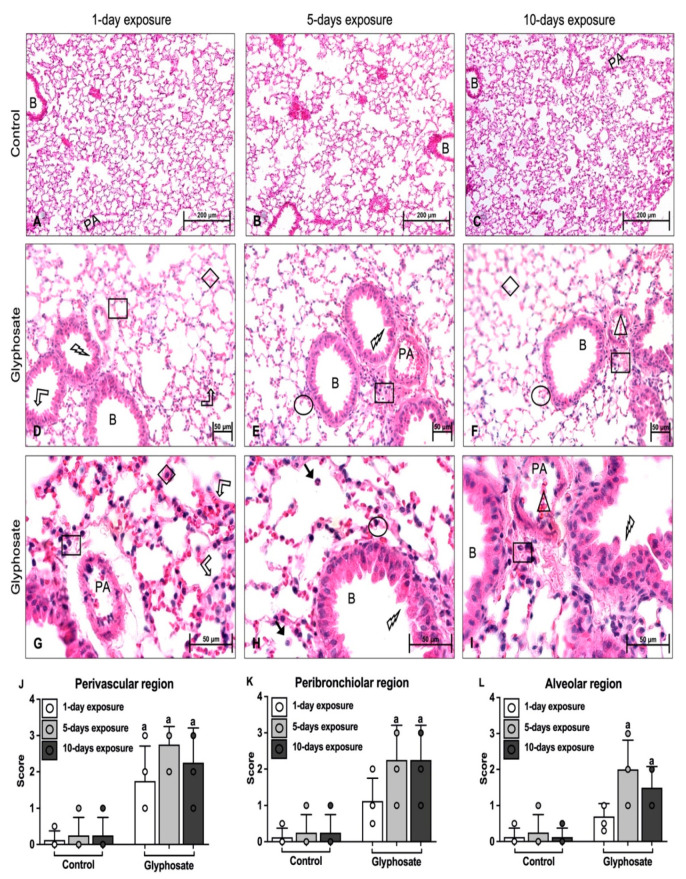
Hematoxylin and eosin-staining of lung sections of mice. Mice lung sections were stained after treatment for 1 day, 5 days, and 10 days with saline (control) (**A**–**C**) or glyphosate (**D**–**I**) and scored for inflammation (**J**–**L**). Group representative images showing perivascular infiltration (indicated by square), peribronchiolar infiltration (indicated by circle), alveolar infiltration (indicated by diamond), perivascular space increase (indicated by double arrow), blood vessel congestion (indicated by triangle), bronchial epithelium thickness increase (indicated by bent up arrow) and sloughing of bronchial epithelial surface (indicated by lightning bolt). Data presented as mean ± SD (**J**–**L**, N = 5 mice per group; 5 fields per section). “a” indicates a significant difference (*p* < 0.05) compared with the respective control group. Magnification: 200 (**A**–**C**); 400 (**D**–**F**); 1000 (**G**–**I**). Scale bar: 200 μm (**A**–**C**); 50 μm (**D**–**I**). PA: Pulmonary artery; B: Bronchus.

Lungs of control mice showed normal architecture without any leukocyte infiltration after 1 day (Figure 4A), 5 days (Figure 4B), and 10 days (Figure 4C) of treatment.

After 1 day of glyphosate treatment, lungs showed occasional sloughing, increased thickness of bronchial epithelium, and slight leukocyte infiltration (Figure 4D,G). There was significantly greater leukocyte infiltration in the perivascular region (Figure 4J) of the 1-day glyphosate-treated animals as compared to that of the 1-day control treatment, whereas there were no differences between the peribronchiolar or alveolar regions (Figure 4K,L).

After both 5 days and 10 days of glyphosate treatment, there was significantly greater leukocyte infiltration in the perivascular (Figure 4J), peribronchiolar (Figure 4K), and alveolar (Figure 4L) regions of the lungs as compared to the 1-day glyphosate treatment, as revealed by the staining in the perivascular, peribronchiolar, and alveolar infiltration, blood vessel congestion, and sloughing of bronchial epithelial surface (Figure 4E,H,I).

### 3.5. ICAM-1, VCAM-1, and vWF Staining

**Figure 5:**
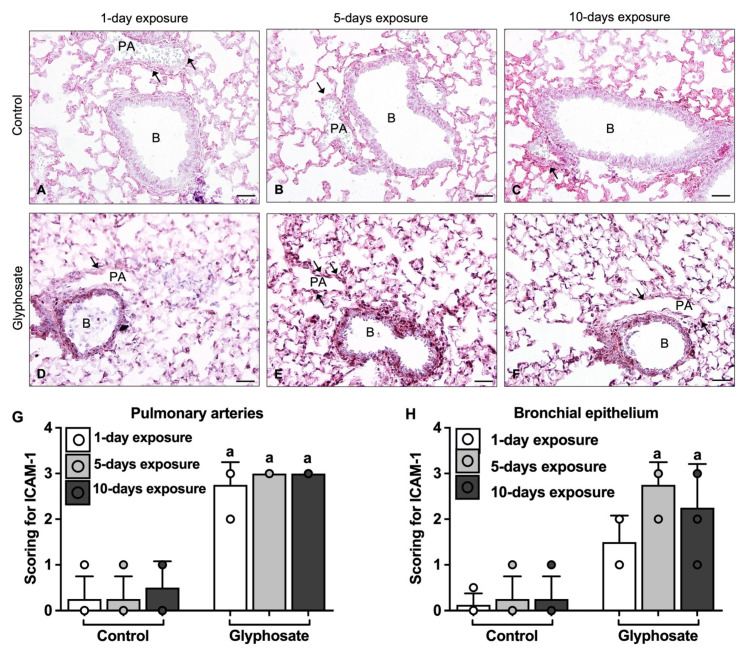
Immunohistochemical expression of ICAM-1 in lung sections of mice after 1 day, 5 days, and 10 days of treatment with control (**A**–**C**) or glyphosate (**D**–**F**) and its scoring (**G**,**H**). Arrow indicates ICAM-1 positive staining in group representative images. Data presented as mean ± SD (N = 3 mice per group; 5 random fields per section). “a” indicates a significant difference (*p* < 0.05) compared with the control group. Magnification: 400 (**A**–**F**). Scale bar: 50 μm (**A**–**F**). PA: Pulmonary artery; B: Bronchus.

Glyphosate treatment for 1 day (Figure 5D), 5 days (Figure 5E), and 10 days (Figure 5F) showed increased staining for ICAM-1 in the alveolar septa and endothelium of large blood vessels. ICAM-1 staining was present in the bronchial epithelium of lungs after all glyphosate treatments, however, it was more strongly stained after 5 and 10 days of glyphosate treatment. Semi-quantification evaluation of the staining further reveals these associations and shows that staining for ICAM-1 was significantly increased in blood vessels after glyphosate treatment for 1 day, 5 days, and 10 days compared to the respective control treatment (Figure 5G). Bronchial epithelium ICAM-1 staining significantly increased after both 5 days and 10 days of glyphosate treatment compared to the control treatments (Figure 5H).

**Figure 6:**
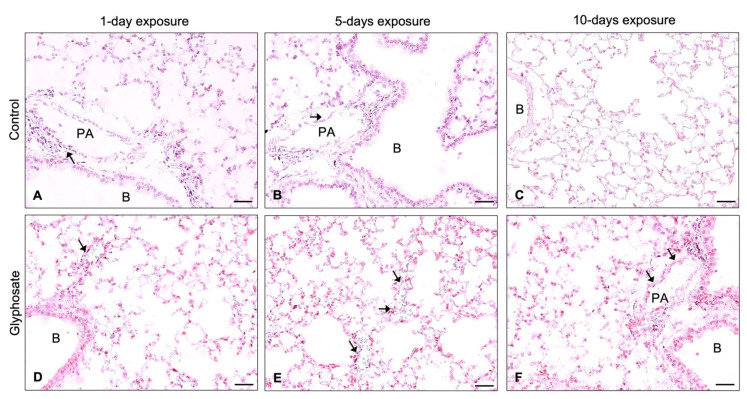
Immunohistochemical expression of VCAM-1 in lung sections of mice after 1 day, 5 days, and 10 days of treatment with control (**A**–**C**) or glyphosate (**D**–**F**). Arrow indicates VCAM-1 positive staining in group representative images. Magnification: 400 (**A**–**F**). Scale bar: 50 μm (**A**–**F**). PA: Pulmonary artery; B: Bronchus.

**Figure 7:**
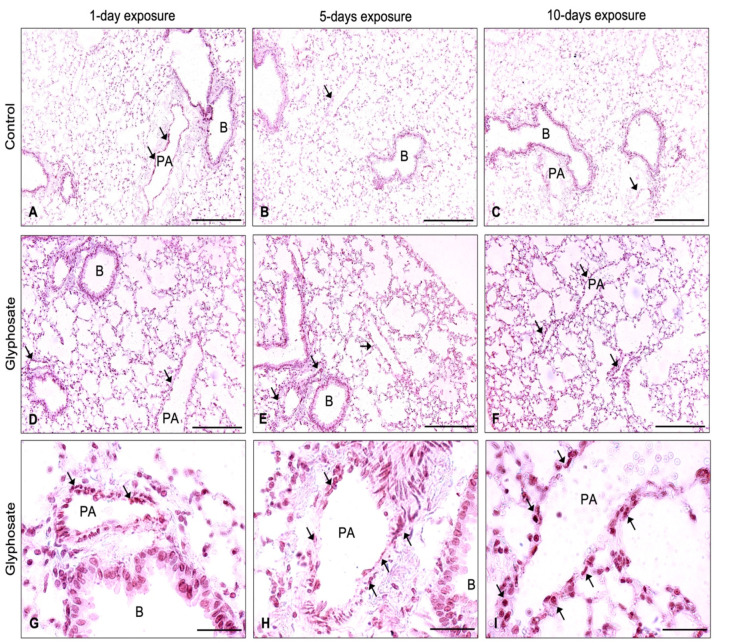
Immunohistochemical expression of vWF in lung sections of mice exposed for 1 day, 5 days, and 10 days to control (**A**–**C**) or glyphosate (**D**–**I**). Arrow indicates vWF positive staining in group representative images. Magnification: 200 (**A**–**F**); 1000 (**G**–**I**). Scale bar: 200 μm (**A**–**F**); 50 μm (**G**–**I**). PA: Pulmonary artery; B: Bronchus.

Similar results were shown for the staining for VCAM-1 (Figure 6D–F) and vWF (Figure 7D–I), revealing staining in the bronchial epithelium, alveolar septa, and endothelium of large blood vessels in lung sections of glyphosate-treated animals for 1 day, 5 days, and 10 days. Lungs of control mice showed positive staining in large blood vessels and minimal staining in the bronchial epithelium and alveolar septa regions for adhesion molecules ICAM-1 (Figure 5A–C), VCAM-1 (Figure 6A–C), and vWF (Figure 7A–C).

### 3.6. ICAM-1, TLR-4, and TLR-2 mRNA

**Figure 8:**
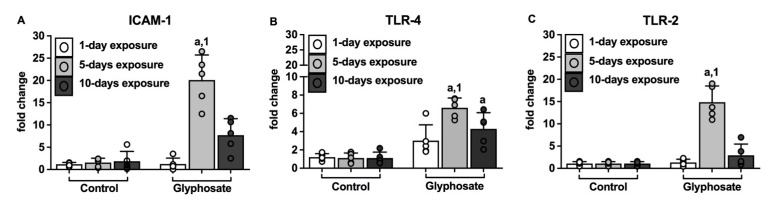
Real time PCR for expression of ICAM-1, TLR-4, and TLR-2 in lungs. Fold change expression of ICAM-1 (**A**), TLR-4 (**B**), and TLR-2 (**C**) in lungs of mice after treatment with saline or glyphosate for 1 day, 5 days, and 10 days. Data presented as mean ± SD (N = 5 mice per group). Significance (*p* < 0.05) is denoted as such: “a” indicates a significant difference compared with the control group; “1” indicates a significant difference compared with 1-day treatment group.

Glyphosate treatment for 5 days significantly increased the levels of ICAM-1 mRNA as compared to the 5-days control treatment and 1-day glyphosate treatment (Figure 8A).

Glyphosate treatment for 5 days and 10 days resulted in a significant increase in both TLR-4 and TLR-2 as compared to the respective control treatment (Figure 8B,C); further, TLR-4 and TLR-2 mRNA levels were significantly higher after 5 days of glyphosate treatment as compared to the 1-day glyphosate treatment. After 10 days of glyphosate treatment, TLR-4 mRNA levels were lower than the 5-day levels but remained significantly higher than the 1-day glyphosate treatment levels (Figure 8B); however, for TLR-2 the 10-day levels were similar to the levels found after 1 day of glyphosate treatment (Figure 8C).

## 4. Discussion

Our results show that repetitive treatment to glyphosate induced greater cellular infiltration in lungs, migration of neutrophils and eosinophils, release of Th2 cytokines, and ICAM-1, VCAM-1, and vWF staining in the alveolar septa and endothelium of large blood vessels, with greater adhesion molecule staining in the bronchial epithelium with increasing days of glyphosate treatment. These results are the first to show that adhesion molecules may be important in leukocyte migration from repeated glyphosate exposure.

The repetitive treatment with glyphosate for 10 days resulted in significantly higher levels of Th2 cytokines IL-5, IL-4, and IL-13 as compared to control treatments. Both IL-5 and IL-13 have important roles in lung inflammation associated with asthma, COPD, and chronic rhinitis [26,27,28]. IL-5 is involved in the recruitment of eosinophils in the asthmatic lung, and we noticed an increase in eosinophils in the lung tissues from glyphosate-treated mice. Eosinophils are also the source of profibrotic factors such as IL-13 [29]. After repeated treatments with glyphosate (10 days, but not 5 days), there was also a significant increase in IL-33 levels in BAL. It has been shown that through the activation of IL-5 and IL-13 secretion, IL-33 promotes airway hyperresponsiveness [30,31]. These Th2 cytokine results are similar to the findings of others reported after 7 days of glyphosate exposure [2] in female mice, in which the work focused on the role of IL-13 in glyphosate exposures. The IL-13 deficiency had no effect on IL-10, prevented a rise in IL-5, but had no effect on IL-33 with longer glyphosate exposure time [2], suggesting a continued role for IL-33 in lung pathology in glyphosate exposure, in the absence of IL-13. Our results show that IL-13 progressively increased with increasing days of repetitive exposure of glyphosate, whereas IL-33 took until 10 days of repeated treatment with glyphosate before a significant rise occurred. Further, our results showed an increase in IL-10 with repeated glyphosate exposure. The repeated glyphosate treatment resulted in damage to the lung epithelium as observed in H&E-stained histological images. The damage to lung epithelium could be the source of induction of IL-33 and IL-10 cytokines [32,33]. IL-33 has been shown to be involved in allergic airway responses [34,35] and with tissue damage by innate pathway Th2 induction [36]. Differentiating the role of cytokines in glyphosate-induced airway inflammation is an important area for future research.

The activation of the pulmonary endothelium by cytokines leads to the expression of adhesion molecules which are important for the tissue recruitment of inflammatory cells, such as neutrophils and eosinophils [12,14]. In this work, a single glyphosate treatment revealed expression for ICAM-1, VCAM-1, and vWF adhesion molecules in the perivascular region of lung sections; with repeated treatment (5 and 10 days), adhesion molecule expression was found in the perivascular, peribronchiolar, and alveolar regions of the lungs, while histology revealed pulmonary cellular effects. The increase in cytokines, such as IL-13 and IL-5, likely contributed to the expression of these adhesion molecules. Repeated treatment with glyphosate resulted in increasing levels of leukocytes and, in particular, a strong neutrophilic response, such that after 10 days of treatment with glyphosate neutrophils were almost double the 5-day level. Results also reveal higher eosinophil peroxidase (EPO) levels with glyphosate treatment as compared to control treatments. Eosinophil peroxidase is released by activated eosinophils and acts as a marker for the presence of eosinophils. Our findings also show an increased expression of vWF adhesion molecule in lungs after repeated glyphosate treatments (5 and 10 days). Expression of vWF has been shown to mediate recruitment of platelets which are known to involve in the leukocyte recruitment [37]. Adhesion molecules have been shown to be involved in airway inflammatory effects in asthma and allergic rhinitis [15,16]. The increased endothelial expression of ICAM-1, VCAM-1, and vWF provides molecular evidence of vascular inflammatory processes in the lungs after repeated treatment with glyphosate.

For agricultural crop production workers, glyphosate is often co-exposed with other well- known lung inflammation stimulants, most notably endotoxin (LPS) as a component of organic dust. Grain dust inhalation has been shown to induce neutrophilic inflammation in exposed individuals as well as in animal models and endotoxin (LPS) in the grain dust is often associated with respiratory dysfunction among grain farmers [38,39,40]. Because bacterial molecules activate cells upon binding with TLRs [41], we examined the expression of TLR-4 and TLR-2 in lungs from mice in our experiments and found an increase in their expression following repeated treatments with glyphosate. Glyphosate is a low molecular weight molecule whereas LPS is a pathogen, and as such, different inflammatory pathways are likely initiated with the different treatments. There were both TLR-2 and TLR-4 responses after our repeated glyphosate exposures, however, both waned after 10 days of glyphosate exposure. This is similar to findings by others after LPS exposure, in which TLR-4 expression initially increased but then decreased significantly 36 h after exposure [42]. In this work, the Th2 response to repeated glyphosate treatment was greater than the Th2 response to LPS [21]. Further evaluation of HMGB-1 and surfactant proteins may delineate additional information on the airway inflammatory processes of glyphosate, given that they are associated with both TLR-2 and TLR-4 receptor activities [43,44]. Activation of the TLR receptors is critical for inflammatory signaling resulting in the release of cytokines and the upregulation of endothelial adhesion molecules, which are necessary for leukocyte migration [45]. Strong expression of the adhesion molecules ICAM-1 and VCAM-1 have been shown with other airway exposures including LPS individually [17,18,19,20] and LPS as a co-exposure with glyphosate [21,22]. Recent research has suggested that a history of pathogen insults or allergy appears to be able to induce a trained immunity, centered around the airway epithelium, which appears important to asthma and asthma exacerbation with repeated exposures [46,47,48]. A trained immunity response may be important in explaining the epidemiologic findings of glyphosate exposed workers. Lung function impairments have been observed in pesticide applicators as well as a prevalence of chronic respiratory symptoms [49,50,51]. Glyphosate exposure has been associated with an increased risk of rhinitis and allergic and non-allergic wheeze among pesticide applicators [5,6,7,8,9] and an exacerbation of existing asthma in agricultural workers [10]. The ability of glyphosate to train the airway epithelium to respond to future exposures is an area for future research.

## 5. Conclusions

Repetitive exposure to glyphosate induced an inflammatory cascade including the release of Th2 cytokines and migration of neutrophils and eosinophils, concomitantly associated with increased pulmonary expression of ICAM-1, VCAM-1, and vWF adhesion molecules and TLR-4 and TLR-2 receptors. The increase in pulmonary expression of ICAM-1, VCAM-1, and vWF adhesion molecules could be important for recruitment of inflammatory cells after repeated exposure to glyphosate. The findings further our understanding of the airway inflammatory potential of glyphosate Future studies are needed on longer-term glyphosate exposure to further investigate chronic airway inflammation, which may assist in explaining the respiratory symptoms with repeated exposures in agricultural workers.

## Data Availability

The data presented in this study are available on request to corresponding author.
